# Metabolism and Immunity-Adapted Radiotherapy (M.I.A.R): A Conceptual Framework for Overcoming the Therapeutic Plateau in Clinical Radiotherapy

**DOI:** 10.3390/curroncol33060364

**Published:** 2026-06-17

**Authors:** Georgios Maravelis, Ioannis M. Koukourakis, Pantelis Skarlos, Michael I. Koukourakis

**Affiliations:** 1 Department of Radiation Oncology, Metropolitan Hospital, 18547 Piraeus, Greece; gmaravelis@metropolitan-hospital.gr; 2 Department of Radiotherapy and Oncology, Medical School, Democritus University of Thrace, 68100 Alexandroupolis, Greece; ikoukour@med.duth.gr; 3 Department of Radiation Oncology, Medical School, University of Thessaly, 41334 Larisa, Greece; p.skarlos@yahoo.gr

**Keywords:** radiotherapy, oncogenes, metabolism, immunity, clinical efficacy

## Abstract

Despite technological advances in radiation therapy (RT), improvements in locoregional control of locally advanced disease remain limited, indicating a plateau in RT effectiveness. It is becoming increasingly clear that RT occurs within a dynamic metabolic microenvironment that merges oncogenic activity with metabolic and immune interactions. This includes responses to oxidative stress, regulation of cell death and survival signals, energy metabolism, protein synthesis, autophagy of molecules and organelles, and ultimately, the anti-tumor immune response. Each tumor, regardless of its histology, maintains a unique molecular and microenvironmental identity that influences its response to RT. Furthermore, RT acts as a cellular stressor that activates responses in cancer and stromal cells, impacting clinical outcomes. The concept of Metabolism and Immunity Adaptive Radiotherapy (M.I.A.R) recognizes that RT success depends not only on radiation dose and distribution but mainly on key interventions that alter and influence the biological environment before, during, and after therapy. It highlights the importance of an initial diagnostic workup, which is achievable with current tools, to identify tumor-specific oncogenes, metabolic, and immune profiles. Within the context of M.I.A.R., effective RT requires tumor preconditioning combined with concurrent use of drugs, including metabolism-targeting agents, to increase tumor sensitivity to radiation. Post-RT metabolic and immune interventions are essential for complete tumor eradication. This involves combining existing oncogene-targeting therapies with available immune treatments, supported by low-toxicity modulating drugs/agents with demonstrated preclinical activity against specific molecular and microenvironmental features. Overall, while MIAR remains a theoretical approach, existing preclinical and recent clinical data, e.g., those exploiting tumor hypoxia and re-oxygenation status, or post-RT immunotherapy, strongly support further dedicated investigation.

## Simple Summary

Despite technological advances in radiation therapy (RT), improvements in locoregional control for advanced disease remain limited. RT occurs within a dynamic microenvironment that integrates oncogenic activity with tumor-specific metabolic and immune interactions. RT acts as a stressor, triggering responses in cancer and stromal cells that drive therapeutic resistance and influence outcomes. The concept of Metabolism and Immunity Adaptive Radiotherapy (M.I.A.R) recognizes that RT success depends not only on radiation dose and distribution but mainly on key interventions that alter the biological environment before, during, and after therapy. Within M.I.A.R., effective RT should be based on an initial diagnostic molecular and microenvironmental characterization of the tumor, which will guide a tumor preconditioning phase, followed by sensitized RT with appropriate drugs and metabolism-targeting agents. Post-RT metabolic and immune interventions are crucial for complete tumor eradication.

## 1. Introduction

Radiotherapy (RT) has achieved a high level of technical accuracy through the years. Advances in imaging, treatment planning algorithms, motion management, and delivery systems have enabled highly conformal dose distributions with better protection of surrounding healthy tissues. From a physical and geometric perspective, modern RT has largely answered the question of where and how the dose should be delivered to the target.

Despite this progress, long-term local and regional control remains limited in locally advanced disease, and several biologically aggressive cancers remain incurable with RT. Tumors such as glioblastoma, pancreatic ductal adenocarcinoma, and oncogene-driven non-small cell lung cancer (NSCLC) are highly resistant to radiation and often recur within the treated areas, even when modern technology and treatment guidelines are followed. Tumor regrowth frequently occurs within the high-dose region, indicating that simply improving geometric precision or increasing the dose uniformly is unlikely to overcome inherent biological resistance.

The cytotoxic effect of RT involves oxidative stress and the induction of DNA damage through free radicals. After each RT fraction, cancer cells need to repair DNA strand breaks to survive. Although some cancer cells undergo apoptosis and mitotic catastrophe after irradiation, certain clones with stem-cell properties increase the activity of various molecular pathways in response to treatment [1]. Cancer cell clones can strengthen their antioxidant defenses, enhance DNA repair, and adapt substrate utilization, which collectively help reduce radiation-induced damage and promote recovery [2,3].

Radiation-induced stress is often mitigated by pre-existing metabolic and stress-adaptive mechanisms, as well as by the enhancement of baseline metabolic and survival pathways [4]. In tumors already predisposed to these programs, RT may accelerate the emergence of increasingly resilient metabolic states during treatment. Adaptive cancer cell biology may better explain tumor resistance and progression than inadequate dose delivery. Supporting this idea, a clinical report from Memorial Sloan-Kettering Cancer Center in NY found that patients with oropharyngeal cancer who showed increased oxygenation on 18F-fluoromisonidazole PET during RT had a 95% loco-regional control rate at 2 years when receiving 30 Gy rather than 70 Gy [5].

The recent success of immunotherapy (IO) in clinical oncology revives a long-standing idea first recognized in the early 20th century. A strong immune system can eliminate irradiated tumors at doses 2–3 times lower than would be necessary without an immune response [6]. Tumor metabolism and microenvironmental conditions—both defined by genetic and molecular events that are abnormally or reactively activated in cancer cells and the associated tumor stroma—have emerged as new targets for therapeutic modulation within the framework of IO. This approach has already demonstrated significant RT potentiation in clinical trials for lung, esophageal, cervical, and rectal cancer [7,8,9,10,11]. The removal of irradiated cancer cells by immune cells after RT completion, including resistant clones, is vital. This process can result in tumor elimination with RT doses that are not explicitly radical, but instead modulate the immune response.

Today, it is evident that technological breakthroughs in radiation therapy have yielded only marginal improvements in locoregional control of locally advanced disease, suggesting a plateau in RT effectiveness. The proposed term in the title of this article, ‘Metabolism and Immune Adapted Radiotherapy’ (M.I.A.R.), describes the need to develop a framework that links technical accuracy with the biological pathways of radioresistance. M.I.A.R. conceptualizes RT as a treatment delivered within a dynamic metabolic environment by integrating information on oncogenic activity with an interactive metabolic background. This background covers the oxidative stress response, regulation of cell death and survival signaling, energy metabolism, protein synthesis, autophagic turnover of molecules and organelles, and ultimately, the anti-tumor immune response. Instead of focusing on increasing the radiation dose to eradicate the tumor, it examines how RT can be delivered within modulated biological conditions that suppress survival pathways and enhance anti-tumor immune responses.

## 2. From Oncogenic Drivers to Functional Metabolic Axes

Over the past decade, routine molecular profiling has increasingly become part of cancer care. Oncogenic alterations are now identified at diagnosis. Recurrent drivers such as *KRAS*, *TP53*, *MYC* amplification, *STK11/LKB1* loss, and *KEAP1/NRF2* pathway activation are routinely used as predictors of survival, response to therapy, and the selection of highly effective targeted treatments [12]. These oncogenes control cellular metabolism and stress response. While they operate through various molecular pathways, they frequently converge on well-known cellular pathways that are directly involved in RT response.

These genetic effects and the extensive molecular cascade they initiate result in functional changes that promote five specific cellular adaptations to survive RT damage:

i. Buffering radiation-induced oxidative stress;
ii. Providing adequate energy for damage repair;
iii. Supplying biomolecular substrates for DNA synthesis and repair;
iv. Securing metabolic adaptation to microenvironmental conditions;
v. Escaping from immune surveillance.

Different oncogenic alterations may therefore converge on similar functional mechanisms that promote radio-resistance. This convergence explains why tumors with high biological diversity frequently show similar patterns of failure within the RT field.

## 3. Metabolic Pathways Underlying Radiation Response

Table 1 summarizes the key metabolic pathways active in cancer cells, which can be targeted to convert the stress effect of RT into direct cell death.

### 3.1. Oxidative Stress Response

Cancer cells can upregulate molecular pathways to reduce oxidative stress caused by RT and prevent cell death [13]. Antioxidant genes that neutralize reactive oxygen species (ROS) include superoxide dismutase (SOD), glutathione peroxidase (GPX), glutathione synthetase (GSS), catalase (CAT), and heme oxygenase (HMOX1).

Activation of this antioxidant pathway is directly controlled by the *Keap1* gene. *Keap1* is part of the E3 Ligase complex that binds to the Nrf1 transcription factor, promoting its proteasome-mediated degradation and subsequent suppression of Nrf1 antioxidant activity [14]. ROS induced by RT modify cysteine residues of *Keap1*, leading to increased cytoplasmic Nrf1, which translocates to the nucleus to bind to antioxidant response elements (AREs) of antioxidant genes, promoting transcription [15]. Deletion of the *Keap1* gene is associated with RT resistance in squamous cell lung and esophageal cancers [16]. Nrf2 inhibitors are currently under investigation. Drugs like brusatol and halofunginone inhibit its translation, while small molecules like ML385 and AEM1 can inhibit Nrf1’s transcriptional activity [17].

The thioredoxin system, composed of thioredoxin, NADPH, and thioredoxin reductase (TRXR), also regulates oxidative stress. Thioredoxin functions as a potent antioxidant by neutralizing free radicals and also regulates the expression of NF-kB and AP-1 [18]. TRXR has emerged as an important pharmacological target for radiosensitization [19]. TRXR inhibitors, such as autanofin, ethaselen, curcumin, and indolequinone, have demonstrated notable radiosensitizing effects [20].

### 3.2. DNA Damage and Apoptosis

RT-induced free radical formation and DNA strand breaks initiate DNA repair mechanisms and also activate p53-mediated apoptosis [21]. Overexpression of DNA repair enzymes like topoisomerases and endonucleases contributes to radioresistance, while deficiencies in mismatch repair genes or mutations in *POLE* (DNA polymerase epsilon catalytic subunit) indicate radiosensitive tumors [22,23,24]. Camptothecins, like irinotecan and topotecan, are topoisomerase I inhibitors, while doxorubicin and etoposide target topoisomerase II [25]. Arylstibonic acids and ML199 analogues are potent inhibitors of human Apurinic/Apyrimidinic Endonuclease 1 (APE1) [26,27]. PARP (poly-ADP ribose polymerase) inhibitors, like olaparib, which inhibit DNA repair, may also be important in the efficacy of RT [28].

*P53* mutations are directly associated with resistance to RT in many human cancers, including head-and-neck cancer, endometrial cancer, and others [29]. Restoring wild-type *p53* function using small molecules such as APR-246 (Eprenetapopt) or reactivating the pathway by inhibiting p53’s interaction with MDM2 with RITA (Reactivating p53 and Inducing Tumor Apoptosis small molecule) could be crucial for patients with p53 mutations [30,31]. The recent failure of RT combined with inhibitors of ‘inhibitors of apoptosis’ proteins (cIAP, XIAP) in head–neck cancer does not rule out an important role for these drugs in specific patient subgroups that overexpress these anti-apoptotic molecules [32]. Targeting the Bcl-2 protein, which inhibits mitochondrial apoptosis, is also a promising approach with an established role in the treatment of hematological malignancies (Venetoclax, Sonrotoclax) [33,34].

### 3.3. Glycolysis and Oxidative Phosphorylation

Oxidative phosphorylation is the main way the human body produces ATP. However, in cancer cells, this process is less active. Instead, they mainly generate energy through conversion of pyruvate to lactate via lactate dehydrogenase A (LDHA), which allows for faster ATP production [35]. Glycolysis also produces several carbon skeletons used in lipid, nucleotide, and amino-acid synthesis [36]. This metabolic shift toward the so-called aerobic glycolysis, or Warburg effect, is mainly driven by the overexpression of Hypoxia-Inducible Factors HIF1α and HIF2α. This overexpression results from either reduced proteasomal degradation or oncogene-driven increases in transcription or translation, involving genes such as *c-myc*, *p53*, *EGFR*, and *ras/BRAF* [37]. Stabilization of HIF may also happen via oncogenic viruses (HPV and EBV) or through abnormal activation of the PI3K/AKT/mTOR pathway [38]. Therapeutic targeting of HIF1α concentrates on the central aspects of metabolism, where diverse oncogenes and tumor-driven processes converge. These encompass glycolysis, angiogenesis, stemness, proliferation, epithelial–mesenchymal transition, invasion, apoptosis, redox balance, and pH regulation, such as LDHA and carbonic anhydrase CA9 [39,40]. All these pathways are directly linked to minimizing the lethal effects of RT, creating a framework for therapeutic strategies that consider radiation exposure as a type of metabolic stress [41]. Overexpression of HIF, CA9, and LDHA has been associated with resistance to RT in tumors such as head and neck cancer [42,43]. Specific HIF inhibitors like PX-478, chetomin, and niclosamide have been identified and demonstrated to have notable radiosensitizing effects [44,45]. Similarly, inhibitors of LDHA activity, such as GSK2837808A, FX11, and Oxamate, as well as natural compounds like Galloflavin and flavonoids, are available to block glycolysis [46]. CA9 inhibitors such as SLC-0111, methazolamide, acetazolamide, and others are also available and have demonstrated radiosensitizing effects [47].

Glucose transporters (GLUTs), genes regulated by HIFs, are also important pumps on cancer cell membranes. They absorb glucose at high rates to support aerobic glycolysis. Targeting GLUTs with small inhibitory molecules (WZB117, BAY-876, Pan-GLUT inhibitor DRB18, HY-139605) is another therapeutic option currently under investigation [48]. Sodium-glucose cotransporter proteins, like SLC5A1 and SLC5A2, move sodium and glucose together across cell membranes. Specific inhibitors (e.g., canaglifozin, dapagliflozin), on the other hand, block active glucose uptake and suppress HIF activity, suggesting a potential role in metabolic interference for cancer therapy, including RT [49]. Dichloroacetate, an orphan drug used to treat children with mitochondrial disorders, inhibits PDH kinase, has synergistic effects with HIF inhibition, and increases cancer cells’ sensitivity to radiation [50].

Blocking aerobic glycolysis, however, may lead to reactivation of mitochondrial activity and oxidative phosphorylation (OXPHOS). Several tumors may also maintain a high baseline OXFOS activity or even exhibit a reverse-Warburg effect, in which anaerobic glucose utilization is facilitated by the tumor-supporting stroma. Inhibition of pyruvate dehydrogenase with drugs like devimistat blocks pyruvate entry into the Krebs cycle for OXPHOS, and may play an important role in the metabolic treatment of cancer [51]. Metformin, an anti-diabetic medication, inhibits mitochondrial complex I, reducing ATP production via OXPHOS, and suppresses the mTORC1 pathway, decreasing protein and lipid synthesis. Although randomized trials have shown no significant effect on breast cancer prognosis, its role in the context of M.I.A.R warrants clinical evaluation [52,53].

Molecular and biochemical targeting of glycolysis with available agents should be individualized following an initial metabolic profiling, as not all glycolytic tumors share the same activated pathways. This should be thoroughly taken into account by ongoing phase II clinical trials, as drug efficacy is expected only in tumors bearing the molecular target.

### 3.4. The Pentose Phosphate Pathway

A pathway closely linked to glycolysis is the pentose phosphate pathway (PPP), also known as the phosphogluconate pathway or hexose monophosphate shunt [54]. Glucose is phosphorylated by hexokinase to G-6-P, a common step in glycolysis and the PPP. The conversion of G6P to NADPH by G6P-dehydrogenase in the PPP is a critical step for cancer cell biosynthesis. NADPH is a reducing agent involved in antioxidant defense and is necessary for synthesizing nucleotides, fatty acids, and cholesterol. It is also a major pathway for the de novo synthesis of ribose-6-phosphate, which is essential for the biogenesis of nucleic acids (the basis for DNA replication and repair), as well as the synthesis of ATP, NADH, and coenzyme A. It is also vital for synthesizing aromatic amino acids such as phenylalanine, tyrosine, and tryptophan.

Regarding the oncogenes involved in PPP regulation in cancer, hexokinase, which directs glucose into the pathway, is upregulated by the *Ras/BRAF* oncogenes and also by an overactive *Wnt/β-catenin* pathway [55]. *C-myc*, *mTORC1*, *Nrf2/EGFR*, and *Src* also contribute to increasing PPP-related enzymes, such as transketolase, transaldolase, and G6PD [56].

Targeting the G6PD appears to be important for cancer cell proliferation and redox homeostasis. It is overexpressed in tumors, especially those that have developed multidrug resistance [57,58]. Dehydroepiandosterone (DHEA), which can be taken as an oral supplement with low toxicity in humans, is a snail repressor that reverses NADPH homeostasis. Although associated with overcoming chemoresistance to taxanes [59], there are no data on radiosensitization. Polydatin, a precursor of resveratrol that acts as a G6PD inhibitor, increases ROS and induces apoptosis, making it a potential strategy to overcome drug resistance and inhibit tumor growth. It also enhances the effectiveness of RT while simultaneously safeguarding healthy tissues from radiation damage [60]. Inhibiting nucleotide synthesis is crucial in preventing cancer cell repopulation during RT [61].

### 3.5. Aminoacid Metabolism

The metabolism of certain amino acids may also be important in radioresistance and could become targets for metabolic interference. Glutamine, the most plentiful amino acid in the blood, is utilized for energy production by rapidly dividing cells (anaplerosis), including cancer cells [62]. Following its conversion to glutamate and α-ketoglutarate, it enters the Krebs cycle to generate ATP, NADPH, and FADH2. α-KG also activates the mTOR pathway. Glutamine provides building substrates for the de novo synthesis of purines and pyrimidines, contributing also to redox balance, acting as a substrate for glutathione (GSH) synthesis, the most important intracellular antioxidant. Blockage of glutamine metabolism may therefore disrupt essential pathways for cancer cell growth, such as DNA synthesis and energy production [63].

Oncogenes like *c-myc*, *EGFR*, and *Kras* are known to promote glutamine addiction in cancer cells by regulating glutamine transporters and glutaminase (GLS), or by upregulating enzymes involved in converting glutamine to α-ketoglutarate for OXFOS [64].

Inhibition of glutamine uptake can be targeted with GLS inhibitors that block the conversion of glutamine to glutamate or with inhibitors of glutamate dehydrogenase (GLUD) that block the conversion of glutamate to α-ketoglutarate [65]. Additionally, glutamine uptake by cancer cells can be blocked by inhibiting the amino acid transporter 2 (ASCT2/SLC1A5), a specific neutral amino acid transporter that absorbs glutamine, alanine, serine, and cysteine from the extracellular environment [66]. Inhibiting glutamine metabolism makes cancer cells more sensitive to RT [67,68].

Other pathways like the Serine and Glycine Synthesis Pathway (SSP) also play an important role in providing building blocks (serine, glycine) for protein, nucleotides, and glutathione synthesis, as well as in cancer stem-cell maintenance. This pathway is targeted for use in cancer therapy and RT [69,70]. The antidepressant sertraline (an inhibitor of serine hydroxymethyltransferase) has recently been shown to improve RT efficacy in NSCLC [71].

### 3.6. Lipid Metabolism

Lipid metabolism is crucial for cancer cell growth and energy production [72]. Fatty acid synthesis (lipogenesis) produces membranes for new cells, while oxidation of fatty acids (FAO or beta-oxidation) can also be used for energy by generating acetyl-CoA that enters the Krebs cycle. Oncogenes like c-myc and Kras are involved in lipogenesis by upregulating the citrate transporter SLC25A1 and the expression of ACSL1 and FASN synthases [73,74]. HIF upregulation is also involved in lipid metabolism by increasing lipid synthesis, storage, and regulating genes involved in lipid uptake, such as VLDLR [75].

Focusing on lipid metabolism as a radio-sanitization strategy has recently gained attention. Chemotherapy and RT can induce lipid-dependent metabolic changes and support the development of resistant cancer cell phenotypes [76]. BMS303141, an ATP Citrate Lyase (ACLY) inhibitor, has radiosensitizing properties [77].

### 3.7. Autophagy

The autophagic machinery carries out a fundamental process of recycling damaged proteins, membranes, and organelles in the cell. These are broken down into ‘building blocks’ for use in anabolic processes and energy production [78]. Autophagy is triggered by fasting and different stress conditions, such as hypoxia, oxidative stress, DNA damage, heat, and viral infections [79]. Initiation of autophagy by the ULK1 complex and subsequent recruitment of Atgs and MAP1LC3 proteins are regulated by multiple genes and oncogenes. Hypoxia, a common physicochemical condition in the tumor microenvironment, is the main driver of autophagy involving HIFs. Mutations of *c-Jun* and *Ras* and activation of *Akt* or *Liver Kinase B1* and normal *p53* function are also positive regulators of autophagy [80,81]. mTOR and BCL-2 have been shown to inhibit autophagy, while *p53* mutations play a more complex role.

The intensification of autophagy characterizes radio-resistance in many tumors, including prostate and lung cancer [82,83,84]. Blocking autophagy enhances tumor response to RT [85]. Similarly, blocking the endoplasmic reticulum stress protein—Glucose-Regulated Protein 78—which positively regulates autophagy, inhibits radioresistance in nasopharyngeal cancer [86]. Silencing *c-Jun* activates the PI3K/AKT/mTOR pathway, which inhibits autophagy and decreases radioresistance in nasopharyngeal cancer [87]. Blocking the fusion of autophagosomes with lysosomes increases the radiosensitivity of glioblastoma cells [88]. A glycogen synthase kinase 3 inhibitor GSK-3beta blocks autophagy and enhances the radiosensitivity of NSCLC [89]. Chloroquine, an antimalarial drug that inhibits autophago-lysosomal fusion, has demonstrated modest radiosensitization in clinical trials for primary and metastatic brain tumors [90]. Potent ULK1/2 inhibitors have recently been developed (e.g., SBI-0206965, ULK-101, MRT68921), and results from their combination with RT are awaited [91,92].

A specific type of autophagy is lipophagy. In this process, lipid droplets, dynamic organelles that store intracellular triglycerides and cholesterol, are engulfed in autophagosomes for subsequent degradation to obtain energy and building blocks [93]. Perilipins are proteins of the lipid droplet membrane and mark them for degradation. Targeting this process sensitizes prostate cancer cells to RT [94].

## 4. Stemness and Senescence as Targets for M.I.A.R

During and after RT, cancer stem cells (CSCs) are recruited into the cell cycle to replace cells lost to RT-induced cell death. Up-regulation of signaling pathways such as *Notch*, *Hedgehog*, *Hippo*, and *WNT* is common in CSCs [95,96]. RT promotes the growth of cancer cell clones that express stem cell markers such as CD44, CD133, OKT4, and others [97,98]. These clones exhibit low ROS levels, high autophagic activity, and a highly glycolytic metabolism, although they can also switch to OXPHOS for energy production [99]. Clinical studies have demonstrated that CSCs are particularly resistant to radiation [100]. Targeting CSCs through signaling pathway inhibition could be essential for interrupting a key tumor survival mechanism during RT. Hedgehog inhibitors such as vismodegib and sonidegib are already well established for treating basal cell skin cancer [101]. Small-molecule inhibitors of the *WNT/β-catenin* pathway have been developed and have entered phase I/II trials [102]. Targeting the *Notch* signaling pathway with monoclonal antibodies against delta-like ligands is also under evaluation in clinical trials [103]. Immunotherapeutic approaches targeting CD133 with CAR T cells, bispecific antibodies, or small-molecule inhibitors such as salinomycin and pyrvinium are also under investigation [104].

Irradiated cells may also follow a different pathway, entering senescence, a state of permanent dormancy in the most radioresistant G0 phase [105]. Although these cells do not proliferate, they remain metabolically active and develop the so-called senescence-associated secretory phenotype (SASP). These cells exert strong bystander effects by releasing cytokines, chemokines, and growth factors that drive chronic inflammation, which impairs anti-tumor immunity [106]. Senolytic drugs that specifically target and eliminate senescent cells could play a key role in lowering the radiation-induced increase in senescent cell content within irradiated tumors. Dasatinib, a tyrosine kinase inhibitor, combined with the natural flavonoid Quercetin, provides powerful senolytic effects [107]. Targeting cIAP2 (cellular inhibitor of apoptosis protein 2) with SMAC mimetics like birinapant eliminates senescent cells in glioblastomas during RT [108]. Bcl-2 inhibitors like Navitoclax, currently in phase III trials for myelofibrosis, also have senolytic activity [109]. Fisetin, another natural flavonoid, is also under investigation [110].

## 5. Metabolic and Immune Pathways Underlying Post-Irradiation Tumor Clearance

Immediately after RT completion, it is uncommon for the tumor to have disappeared clinically or radiologically. In clinical practice, responses are usually assessed with CT or MRI after at least two months, allowing time for the tumor to shrink. During this period, reproductive cancer cell death and gradual depopulation may reach the optimal endpoint of complete eradication or lead to less favorable outcomes, such as complete clinical remission with residual subclinical disease or tumor regression with clinically evident disease. In these latter two cases, the tumor reaches a balance between growing and dying cancer cell clones, supported by an additional compartment of quiescent and senescent cancer cells. Usually within a few months, this balance shifts in favor of tumor progression. Modulating the antitumor immune response is crucial for destroying actively growing cancer cells and stem cells, thereby shifting the balance toward tumor elimination.

RT induces key molecular events in cancer cells, leading to the activation of dendritic cells within the tumor and the subsequent education and activation of cytotoxic T-cells in regional lymph nodes. The induction of the IFN-type-I response and the release of IFNα, IFNβ, and downstream proteins are key to the radiation-induced activation of the anti-tumor immune response [111]. Restoration of HLA-class I expression by cancer cells through RT further improves their recognition and destruction by activated T-cells [112]. This primed condition in the irradiated tumor, known as “radio-vaccination,” creates a favorable environment for immune cells to eliminate cancer cells. Residual clinical or subclinical disease after RT becomes a more vulnerable target for the immune system. This hypothesis is now well supported by clinical trials involving post-RT IO in lung, cervical, esophageal, and rectal cancers, as reported above. The metabolic pathways discussed earlier directly influence the immune response in the tumor microenvironment, which is essential for the highly sought-after immune-mediated ‘tumor clearance’ [113] (Table 2).

### 5.1. Oxidative Stress Response

ROS generated after irradiating cancer and tumor stromal cells activate the NF-κB pathway and inflammasomes, stimulating innate immune cells such as macrophages and neutrophils. This promotes inflammatory responses that lead to the release of proinflammatory cytokines, including TNF-α, the most potent promoter of cytotoxic responses [114]. The redox status also directly impacts immune cell metabolism, which in turn regulates their differentiation and function, influencing T helper cell and macrophage activation [115]. Chronic oxidative stress, however, leads to T-cell exhaustion in the tumor microenvironment or even activates myeloid cells that suppress T-cell activity [116]. NF-κB inhibitors administered after RT may help reduce oxidative stress and restore the responses of cytotoxic T-cells and macrophages. Proteasome inhibitors like Bortezomib, IKK complex inhibitors, or natural compounds such as curcumin and resveratrol may prevent NF-κB activation and enhance immune responses after radiation therapy [117,118].

### 5.2. Glycolysis

Highly glycolytic tumors create an immunosuppressive microenvironment. Cytotoxic T-cells rely on glucose to remain active and carry out their cytotoxic functions [119], so glucose consumption by cancer cells in the tumor microenvironment competes with immune cell demands, leading to T-cell inactivation [120]. Drugs that block glucose uptake, such as GLUT1 inhibitors and SGLT2 inhibitors, induce a shift in cancer cell metabolism toward oxidative phosphorylation, thereby conserving glucose for T-cells [121].

Up-regulated LDHA in cancer cells increases ATP and lactate production, and both are released into the surrounding tumor stroma. Lactate and the resulting acidification of the extracellular matrix create an unfavorable microenvironment that blocks T-cell proliferation and cytotoxic activity, leading to T-cell exhaustion [122]. Policies that elevate tumor pH are increasingly considered promising options for cancer IO. Glucose uptake inhibitors, LDHA inhibitors, monocarboxylate transporter inhibitors, 2-deoxy-D-glucose, and even dichloroacetate—which shifts metabolism toward oxidative phosphorylation—are currently under investigation [123]. Inhibitors of CA9, such as SLC-0111, an important enzyme that promotes tumor acidification by releasing carbonic acid, may also contribute to this process [124]. Inhibiting glycolysis by disrupting its complex molecular regulation could be vital for destroying irradiated tumors; this may occur either by directly killing cancer cells or by reducing immunosuppression caused by glucose deprivation and acidosis in the tumor microenvironment.

### 5.3. ATP and Adenosine Production

The abundant release of ATP in the tumor stroma during aerobic glycolysis, along with amino acid and lipid metabolism, creates an energy-rich environment that signals danger and promotes T-cell activation via P2 × 7 purinergic receptors [125]. However, overexpression of ectonucleotidases by cancer cells and stromal fibroblasts rapidly metabolizes ATP to AMP and adenosine [126,127]. Adenosine is a powerful immunosuppressive metabolite that inhibits T- and NK cell activity while promoting regulatory T-cell activity [128,129]. Radiation can increase the expression of CD73 ectonucleotidase and adenosine receptors in myeloid suppressor cells [130]. Blocking CD73 and CD39 ectonucleotidases or using adenosine receptor antagonists (e.g., AZ10606120) enhances both local and abscopal effects of RT in experimental models [131,132]. Ectonucleotidase inhibitors might reduce adenosine levels and boost immune-mediated tumor clearance following RT [133].

### 5.4. Amino Acid Metabolism-Related Immunosuppression

Amino acid metabolism can lead to the production and accumulation of potent immunosuppressive molecules within the tumor microenvironment. Enzymes such as indoleamine-2,3-dioxygenase (IDO1) and tryptophan-2,3-dioxygenase (TDO2), which convert tryptophan into the immunosuppressive metabolite L-kynurenine, are highly expressed in human tumors such as breast cancer and are linked to an immunologically cold microenvironment [134]. Targeting IDO1 can reverse this immunosuppression and overcome radioresistance in lung cancer [135]. Kynurenine promotes radioprotection in colon cancer, and this effect is reversed by IDO1 inhibitors [136]. A synergistic effect of IDO1 inhibition, PD-1 blockade, and radiation has been documented in glioblastomas. Blocking IDO1 with indoximod (1-methyl-D,L-tryptophan) [137] enhances T-cell anti-tumor activity and might become a useful partner in RT-driven tumor eradication.

Arginine, a conditionally essential amino acid, is another target for IO. Arginine deprivation induces cell cycle arrest and suppresses the mTOR pathway [138]. Although normal cells can synthesize arginine from citrulline via argininosuccinate synthase (ASS1), approximately 25% of tumors lack ASS1 and are auxotrophic, relying on external arginine obtained from the tumor stroma for growth [139]. Consequently, T-cells infiltrating the tumor encounter an arginine-depleted microenvironment that hampers their proliferation and the sustained expression of T-cell receptors required to bind cancer cells [140]. Although arginine deprivation therapy may directly kill autotrophic tumors, it also reduces T-cell cytotoxicity. Agents that enhance ASS1 activity, such as spinosine A derivatives, may restore ASS1 expression in auxotrophic tumors and create an environment conducive to tumor-infiltrating cytotoxic T cells, an important step in immune-mediated clearance of the irradiated tumor [141].

### 5.5. Autophagy and HLA-Class-I Expression

Complete or extensive loss of HLA-class I expression is common in cancer [142,143]. Suppression of HLA-class I usually results from a coordinated process that reduces the activity of HLA, β2-microglobulin, and antigen transporter genes. Therapeutic strategies to restore HLA-class I expression lost in NSCLC could improve the effectiveness of ICIs and help eliminate irradiated tumors. Recent experimental data indicate that autophagy suppresses HLA-class I expression and helps tumors evade immune surveillance [144].

Yamamoto et al. demonstrated that autophagy-driven degradation of MHC class I is a crucial mechanism by which pancreatic cancer escapes immune detection, and that chloroquine can restore anti-tumor immune responses [145]. In endometrial cancer, the autophagy protein LC3 binds to and inhibits the MHC class I transactivator NLRC5, repressing MHC expression and facilitating immune evasion [146]. Autophagy inhibitors such as chloroquine may restore HLA class I expression and enhance T-cell cytotoxic activity [147].

## 6. Timing of Metabolic and Immune Interventions

The role of chemotherapy with DNA-damaging agents and antimetabolites is well established for several locally advanced tumors, as clinical trials have confirmed the importance of concurrent administration throughout the course of RT. A key challenge in modern immuno-RT is determining whether IO should be administered before, during, or after RT, as the optimal timing remains uncertain. The immunotoxic effect of RT, which is exerted at several levels, including the local tumor, regional lymph nodes, and systemic radio-induced lymphopenia, is certainly a major parameter that should be taken into account. A similar question may arise for all types of metabolic manipulations. Timing is essential to the clinical planning of M.I.A.R. Interference with metabolic and immune functions should be viewed as a long-term regulatory biological intervention. The individualized molecular and biological therapy necessary for RT success should be well understood through thorough diagnostic characterization. Interventions should be designed to target cancer cells, microenvironmental adjustments, radiosensitization, and the post-RT remnant disease.

**Figure 1:**
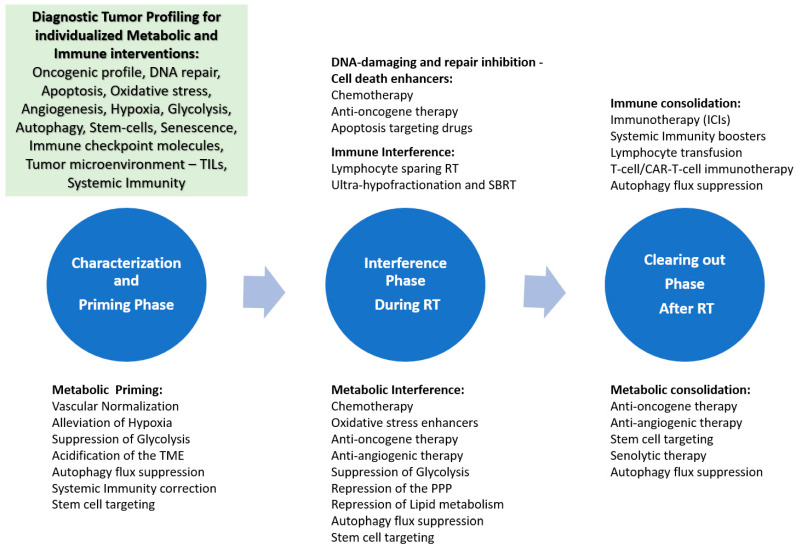
Overview of tumor assessment and therapy during the three distinct stages: i. ‘characterizing’ and ‘priming’ during the preparatory phase before RT, ii. ‘interfering’ during the course of treatment, and iii. ‘clearing out’ after RT.

Briefly, the success of M.I.A.R. depends on sequential phases, beginning with a preparatory phase that characterizes the tumor’s metabolic and immunological features, adjusts cancer cell metabolism, and modifies relevant microenvironmental conditions. During RT, interventions aim to enhance radiation-induced cell death pathways and prevent the emergence of radioresistant cancer cell clones or microenvironmental adaptations that could facilitate post-RT tumor regrowth. Finally, post-RT interventions primarily focus on preventing microenvironmental changes and residual cancer cell adaptations that might hinder the immune system’s ability to effectively eradicate the remaining tumor. Briefly, these three phases of tumor assessment and therapy can be summarized as follows: i. ‘characterizing’ and ‘priming’ during the preparatory phase before RT, ii. ‘interfering’ during the course of treatment, and iii. ‘clearing out’ after RT (Figure 1).

### 6.1. Characterizing and Priming

Patterns of metabolic activity and antitumor immune responses must be identified during disease diagnosis and staging. Characterizing each tumor by its molecular profile of gene mutations, oncogene alterations, and protein overexpression is feasible and increasingly used in clinical practice. Testing for *EGFR/Raf/Ras* and *ERK/MEK* mutations is essential before starting treatment for NSCLC or melanoma. These oncogenes promote a glycolytic metabolic profile and HIF overexpression, leading to a hypoxic and acidic microenvironment. These conditions enhance resistance to apoptosis, increase migration, and generally inhibit RT-induced cell death. Highly effective third-generation tyrosine kinase inhibitors, such as Osimertinib, are already in clinical use and can be used during this ‘priming’ phase of M.I.A.R [148]. The combination of such molecules with RT requires careful investigation [149].

Angiogenic activity is readily assessed by immunohistochemistry through measurement of vascular density and expression of VEGF and its receptors. Overactivity drives invasion, metastasis, intratumoral perfusion hypoxia, and HIF stabilization. These tumors are resistant to RT and can rapidly relapse via ‘angiogenic regeneration’ [150]. Anti-VEGF monoclonal antibodies and VEGF receptor inhibitors are well-established treatments for colorectal, lung, and other cancers. Anti-VEGF therapy induces ‘vascular normalization,’ eliminating immature blood vessels, improving blood flow, and promoting reoxygenation [151]. Anti-angiogenic therapy before RT may therefore improve oxygenation and, when combined with inhibitors of enzymes involved in ROS neutralization or even OXFOS inhibitors, prime the tumor to become highly sensitive to RT. Anti-VEGF combined with RT has demonstrated impressive complete responses in preoperative RT for rectal cancer [152,153]. Vascular normalization during the priming phase aims to remove a key obstacle to RT effectiveness by enhancing re-oxygenation prior to RT. This helps prevent a large portion of the RT dose from being lost in a hypoxic environment, which may or may not be reversed by RT-induced re-oxygenation, potentially occurring after weeks of RT.

Assessment of resistance to apoptotic stimuli is also important. This can be done either through NGS assessment of *p53* mutations or by immunohistochemistry. *p53* mutations can be evaluated in tumor tissue or liquid biopsies (circulating DNA) and are associated with resistance to RT [154]. Expression of inhibitors of ‘inhibitors of apoptosis’ proteins (cIAP, XIAP) and of Bcl2-related proteins can be easily measured by immunohistochemistry on tumor biopsies [155,156,157,158]. A novel radiotracer, 18F-FluorThanatrace ([18F]-FTT), has been developed to clinically assess PARP activity with PET/CT [159]. Although anti-antiapoptotic approaches appear suitable during RT, their value in the priming phase cannot be ruled out.

HIF inhibitors can also help achieve this by reducing aerobic glycolysis and VEGF secretion in cancer cells. Overexpression of HIFs, whether due to decreased degradation under hypoxia or to mRNA overexpression, can be readily detected by immunohistochemistry on tumor biopsies and is strongly linked to resistance to RT [42,160]. In parallel, misonidazole PET-CT can characterize hypoxic tumors, mainly those caused by impaired perfusion, and should be adopted as a standard procedure before RT. The restoration of hypoxia after anti-EGFR, anti-VEGF, or anti-HIF priming therapy can be evaluated using repeated biopsies or misonidazole PET-CT. This approach might enable a significant reduction in RT dose, as recent studies have suggested [5]. In the same context, characterizing the glycolytic profile of cancer cells through immunohistochemical assessment of LDHA, CA9, glucose, and monocarboxylate transporters, or by assessing enzyme overexpression such as hexokinase and others, should be carried out during the characterization phase to guide interventions within the context of M.I.A.R. Overexpression of these enzymes is associated with resistance to RT [161,162]. Using 18F-FDG PET/CT, it is also feasible to assess total lesion glycolysis [163]. Addressing hypoxia and vascular blood flow during the priming phase may prove equally beneficial as expected during the interfering phase.

An overactive autophagic machinery may also be inferred from immunohistochemical assessment of autophagy and lysosomal proteins, such as LC3A and LAMP [164]. PLIN detection associated with radioresistance can also be evaluated in tumor biopsies using immunohistochemistry [94]. Documentation of high autophagic flux could be blocked by autophagy inhibitors already in clinical practice, e.g., chloroquine, during the priming and interfering phase.

Detection of cancer stem cells and senescent cell abundance in the tumor is also important for guiding subsequent phases of M.I.A.R. Immunohistochemistry can detect cancer cells that express stemness markers such as CD44, CD133, and others [100,165]. A novel method, SenTraGorTM, which detects lipofuscin accumulation, can reliably identify senescent cells using a histochemical/immunohistochemical procedure in tumor biopsies [166]. Senolytic drugs are expected to be beneficial during RT, but early initiation of medication in the priming phase may also enhance the benefit.

Characterization of the immune environment can also be performed through histopathological assessment of tumor-infiltrating lymphocyte (TIL) density and immunohistochemical analysis of specific lymphocyte subpopulations, including CD8+ cytotoxic, CD25+ or FOXP3+ regulatory, and NK lymphocytes. Immunologically cold tumors are resistant to RT [167]. A prevalence of regulatory or exhausted CD8+ T-cells may hinder post-RT tumor clearance [168]. PET/CT assessment of TILs is an emerging, noninvasive diagnostic method that uses tracers such as 2′-deoxy-2′-[18F]-fluoro-9-β-D-arabinofuranosylguanine (18F-AraG), which shows high uptake by activated cytotoxic T-cells. Immunohistochemical assessment of immune checkpoint inhibitory molecules (PD-1/PD-L1 or CD80/86) and of enzymes related to amino acid metabolism that predict an adverse immune microenvironment for T-cells is essential to guide targeted IO. Assessment of ectonucleotidases, ASS1 and IDO1/TDO2 is also important and can be performed with immunohistochemistry [139]. All the above become a valuable tool for assessing immunologically hot and cold tumors [169]. Patients with immunologically cold tumors could benefit from priming with autophagy blockers or even from stereotactic large-fraction RT, at least for part of the total prescribed RT dose [145,147]. Hot tumors could benefit from ICIs during the priming phase, as a conspicuous reduction in tumor burden or even complete responses can enhance the outcome of RT, eventually allowing a de-escalation of RT dose. The documentation of hot tumors should also be considered to adjust RT fields and avoid unnecessary irradiation of large areas of regional uninvolved nodes. Immunological priming and interference could be important in supporting SBRT and short-course hypofractionated RT, which have lower systemic lymphotoxic effects [170]

During this characterization phase, assessing systemic immunity via flow cytometry of peripheral lymphocyte subpopulations can inform therapeutic strategies to manipulate the systemic immune response using cytokines or other approaches that promote cytotoxic T-cell activation or suppress regulatory T-cells [171]. Systemic immunity is crucial for the ongoing renewal of cytotoxic T-cells within the tumor microenvironment [172].

Table 3 summarizes the required tumor profile and systemic immunity characterization that will serve as the basis for M.I.A.R.

### 6.2. Interfering Phase

During this phase, the objective is to strengthen RT stress to cause significant cancer cell depopulation, inhibit reactive angiogenesis and autophagy, and ultimately disrupt immune evasion mechanisms. Concurrent chemotherapy with DNA-damaging agents and inhibitors of potentially lethal damage repair, such as cisplatin and 5-fluorouracil, and/or apoptosis and mitotic catastrophe inducers like taxanes, already plays an established role in most human tumors. As described above, blocking ROS-scavenging enzyme systems, inhibiting glycolysis and/or OXPHOS, and using drugs that target the pentose phosphate pathway make cancer cells more vulnerable to RT stress, shifting the balance toward cell death pathways. Similarly, autophagy inhibitors can further enhance the RT stress effect in tumors with high autophagic or lipophagic activity and prevent reactive upregulation of autophagy under RT stress. Anti-angiogenic interference may also be crucial in highly angiogenic tumors that could undergo angiogenic regeneration during RT due to VEGF reactive overexpression.

Based on molecular characterization, RT fractionation and radiation quality can be adjusted accordingly. Documentation of a high proliferation index (MIB1) by immunohistochemistry may suggest the need for accelerated RT schedules, such as a simultaneous integrated boost [173,174]. A hypoxia-indicative profile, suggested by HIF overexpression, low vascular density, or 18F-Misonidazole PET, could identify a subgroup of patients who might benefit from heavy-particle RT [175].

Regarding immunological interference, RT could be used as a radio-vaccination agent. Most tumors show a significant decrease in HLA-class I expression, as previously discussed, and RT should aim to restore the immune system’s recognition of cancer cells. Large RT fractions in the ultra-hypofractionation range (6–8 Gy) strongly induce the type I IFN response, activating dendritic cells and increasing HLA-class I molecules on cancer cells. This effect arises from the direct exposure of cancer cells to IFNα and β produced by the cancer cells, as well as from direct inhibition of autophagy, which reduces the recycling of HLA molecules on the cancer cell surface [176]. In fact, autophagy-targeting drugs like chloroquine are strong inducers of HLA-class I expression, and using them together with RT may improve the restoration of HLA-class I expression [147]. This is extremely important for the subsequent phase of tumor clearance.

Senolytic drugs may also play a crucial role in this phase by reducing the number of senescent cells that gradually accumulate in the irradiated tumor. This helps prevent chronic inflammation in the tumor microenvironment and enhances the immune system’s ability to eliminate cancer during the ‘clearing-out’ phase.

### 6.3. Clearing out Phase

Because RT is immunotoxic, it is essential to re-evaluate the systemic immune response during the ‘clearing out’ phase. Lymphopenia caused by RT is a common side effect, with grade III/IV lymphopenia (<500/μL) occurring in about 50% of patients treated for brain, chest, or pelvic tumors, and its severity is linked to worse outcomes [177]. Lymphopenia following radiochemotherapy in stage III NSCLC patients receiving durvalumab consolidation IO is linked to reduced treatment effectiveness [178,179,180]. Maintaining a robust lymphocyte system is crucial for post-RT elimination of the irradiated tumor. This involves adjusting RT planning to reduce the dose to the bone marrow and organs with high blood flow, such as the heart and major vessels, and considering ‘involved lymph nodes’ rather than ‘elective’ irradiation of regional lymph nodes [181,182]. Ultra-hypofractionated schemes, SBRT, and proton therapy, along with their radio-vaccinating properties, also help maintain adequate lymphocyte counts [183,184]. The use of cytoprotective drugs, lymphocyte-stimulating cytokines, growth factors, or more invasive techniques, such as harvesting lymphocyte subpopulations prior to RT for later re-infusion, should be considered essential clinical strategies [185,186,187,188]. Pre-RT harvesting of CD8 T-cells, their ex vivo activation, and re-infusion after RT may offer an alternative, potentially more effective approach [189]. CAR-T cell technology could play a significant role in the post-irradiation tumor clearance phase [190].

Beyond lymphocyte protection and expansion, several interventions can enhance lymphocyte activity within the tumor during the ‘clearing-out’ phase. Current IO with ICIs (anti-PD-L1, anti-PD-1, anti-CTLA-4) is crucial for overcoming T-cell inactivation caused by cancer cells. This post-RT consolidation IO has already become the standard of care for many human carcinomas, as previously discussed [7,8,9,10,11]. Supporting ICIs with HLA-enhancing drugs (e.g., autophagy blockers) or with personalized interventions to reverse immunosuppressive microenvironmental conditions (e.g., reducing adenosine and kynurenine), implementing policies to prevent T-cell exhaustion using specific monoclonal antibodies (anti-LAG3, anti-TIM3), or even using anti-VEGF therapy to inhibit angiogenic regeneration in highly angiogenic tumors may be essential. Older cytokine-based immunotherapies with IL-2, GM-CSF, or IFNα should also be re-evaluated alongside ICIs during the ‘clearing-out’ phase. Finally, surgical excision or ablative interventions for remaining disease should be included in the M.I.A.R. portfolio.

In the clearing-out phase, senolytic and stem-cell targeting agents may also be important.

## 7. Additional Considerations

### 7.1. Protons and Heavy Particles

Beyond the effects of RT fractionation, the choice of RT modality is equally critical in the context of MIAR. Proton and heavy ion therapy have not been extensively studied when it comes to metabolism and the immune system; however, several reports have suggested effects different from the ones that photons confer. Because their characteristic Bragg peak spares neighboring normal tissues, these modalities inflict significantly less damage on surrounding blood volumes, bone marrow, and unaffected lymph nodes [191,192], allowing for higher tumor lymphocytic infiltration and cytotoxic activity. Moreover, preclinical data have displayed that carbon ion therapy could potentially stimulate a more robust immune response when compared to photon therapy, while also limiting the activation of immunosuppressive cells (e.g., myeloid-derived suppressor cells) [193]. Data on proton therapy are also inconclusive [194,195]. Regarding metabolomics, the existing literature on proton and heavy ion therapy is scarce, and further investigation is required in order to extract safe conclusions.

### 7.2. Safety Assurance

A primary challenge when combining RT with systemic therapy (e.g., targeted agents, ICIs) is the increased incidence of adverse events and the severity of treatment-related toxicity. Nevertheless, extensive data from randomized trials have provided significant experience with these combinations, enabling the development of guidelines on safe intervals between treatments, the feasibility of concurrent administration, and potential drug dose modifications to achieve optimal patient outcomes [196,197,198]. These cover not only conventional RT (both palliative and radical) but also stereotactic approaches, and can provide a strong foundation for future clinical trials evaluating MIAR. Although metabolic inhibitors combined with RT have not yet been extensively studied, upcoming clinical trials will be crucial for identifying any severe or life-threatening adverse events. Because the proposed framework introduces interventions at different stages (before, during, and after RT), rigorous patient monitoring will be imperative to ensure the tolerability and safety of the prescribed regimens. Typical examples of concern about increased radiation toxicity include the anti-VEGF therapy [199] and ICI combinations with RT. Clinical trials, however, have shown good tolerance of anti-VEGF and RT in rectal cancer [151,200], while increased low-grade radiation pneumonitis after ICI/RT does not increase mortality [201]. In addition, the above studies combine molecular interference with standard RT doses, whereas the main aim of a therapeutic approach using the M.I.A.R concept is to decrease RT dose and RT-field parameters, which is expected to reduce both early and late RT sequelae [202].

### 7.3. Cost Effectiveness

A major issue with proposed interventions that include comprehensive gene analysis using next-generation sequencing, numerous immunohistochemical stains, multiple specialized PET/CT scans, and the use of expensive drugs is the expected high healthcare costs. Prices for newer targeted drugs are high, and thus public healthcare systems are called upon to cover those costs, provided that phase 3 trials have confirmed benefits. Financial discrepancies among the populations of countries that predominantly rely on patient insurance to cover hospital expenses can lead to imbalances in healthcare access, which may also apply to the MIAR approach [203,204]. As suggested by Jazowski et al., the financial inability to secure access to advanced cancer treatments can lead to higher rates of disease recurrence and poor clinical prognosis [205]. From the physician’s perspective, an algorithm should be established that allows the implementation of the strictly necessary diagnostic tests, and prioritize interventions when resources are limited. Khan et al. has also advocated for the establishment of “financial navigators” that could contribute to mitigating patient-level financial toxicity [203].

Looking at Table 3, we can see that the major diagnostic/treatment-guiding costs stem from NGS tests already included in clinical practice (clinically established biomarkers). The second tier of well-validated biomarkers in translational research necessary for the clinical evaluation of patients to guide M.I.A.R. is primarily based on immunohistochemical assessment, which is inexpensive and could be further simplified and made readily available through automated multiplex immunofluorescence. The cost of these expanded pre-treatment diagnostics should be considered minimal, provided that M.I.A.R. will increase cancer curability and minimize radiation-induced toxicity. It should be stressed that M.I.A.R. does not aim to simply prolong progression-free survival, but rather to achieve tumor elimination and patient cure, and as such would contribute to a drastic reduction in the overall cost of treatment for patients with recurrent disease.

### 7.4. Tissue Availability

While the prospect of mapping the complete metabolic and immune profiles of each tumor through pathology and immunohistochemistry is very enticing, the fact that, in a significant proportion of cases, only small tumor samples are available via biopsy (before radical treatment) complicates this quest. Moreover, RT and systemic therapy can induce changes in tumor behavior (through alteration of gene expression) and the microenvironment, suggesting the need for additional biopsies after initial treatment strategies. Liquid biopsy can provide significant information for tumor gene profiling and assessment of overall tumor metastatic potential, but cannot serve as a reliable surrogate for the tumor microenvironment. There is ongoing investigation into whether information regarding cellular and matrix composition can be accurately extracted through spatial transcriptomics and/or MRI-based intratumoral and peritumoral radiomics, without the need of invasive procedures [206,207]. Provided that the early steps of M.I.A.R. prove encouraging, the way to obtain the proper biopsy necessary to provide adequate information should become a subject for surgeons and endoscopists to develop and adhere to guidelines according to the cancer type and stage.

## 8. Conclusions

M.I.A.R. offers a clinically feasible platform that combines oncogenic biology, energy metabolism, microenvironmental factors, and anti-tumor immune responses, with RT at its core, to enable personalized cancer treatment. It recognizes that tumors often relapse within properly irradiated areas despite technically perfect RT and accepts the existence of a biological limit beyond which further technical improvements do not provide additional therapeutic benefit. It emphasizes the importance of an initial diagnostic workup, which is feasible with today’s globally available tools, to identify tumor-specific oncogenes and metabolic and immune profiles before implementing personalized treatments at various stages—before, during, and after RT. M.I.A.R considers RT a treatment whose success depends not only on the dosage and distribution of radiation within the body but also primarily on interventions that modify and influence the biological environment before, during, and after treatment. It highlights the importance of pre-conditioning in enhancing tumor sensitivity to radiation and advocates for post-RT treatments to ensure complete tumor eradication. This involves using existing oncogene-targeting drugs along with available immune therapies, flanked by low-toxicity metabolism-interfering drugs with proven pre-clinical activity. This therapeutic adjunct aims to reverse inherent cancer- and microenvironment-related radioresistance, prevent the emergence of resistant cancer clones associated with stemness and senescence, and, finally, modulate the tumor microenvironment and systemic immunity to facilitate immune-mediated tumor eradication.

Identifying molecular and metabolic profiles prior to therapy, and ideally tracking metabolic changes during RT, could greatly transform the delivery of RT to cancer patients. Recognizing tumors that are initially sensitive or likely to respond metabolically—such as those with suppressed autophagy or undergoing re-oxygenation—could enable lower radiation doses, leading to effective treatment with fewer side effects. Conversely, early detection of radio-resistant tumors or those expected to activate molecular pathways of radio-resistance during RT could enable targeted molecular and metabolic therapies—already available—that can transform radiation from a doomed-to-fail stressor into a highly effective therapeutic weapon. The post-RT period is a crucial window for immunological and other interventions to eliminate any remaining or persistent irradiated tumor.

The proposed diagnostic work-up and targeted therapies, based on the key molecular pathways identified in each tumor, lay the groundwork for creating algorithmic treatment strategies. These personalized recommendations could support clinical decision-making for each patient receiving curative RT and surpass the therapeutic limits of the current remarkable RT technology.

## Figures and Tables

**Table 1:** Main metabolic pathways active in cancer cells that can be targeted to turn the stress caused by radiotherapy into direct cell death. Oncogenes/genes involved, pathway endpoints, and drugs for potential therapeutic interventions are included.

Metabolic Pathway	Oncogenes and Genes Involved	Endpoints	Targeting Drugs
Oxidative stress response	SOD, GPX, GDD, CAT, HMOX1, *Keap1*/Nrf1, Thoredocin/TRXR	DNA protection and repair.	Nrf2-inhibitors (e.g., brusatol, halofunginone, and small molecules like ML385 and AEM1). TRXR-inhibitors (e.g., autanofin, ethaselen, curcumin, and indolequinone).
DNA repair	Topoisomerase I and II, HAP1, MMR genes	DNA strand break repair.	Camptothecins. Liposomal doxorubicin. Arylstibonic acids. ML199 analogues. PARP inhibitors.
Apoptosis	P53. Bcl-2, cIAP/XIAP	Inhibition of anti-apoptotic pathways.	APR-246. RITA. cIAP/XIAP inhibitors—Xevinapant. Venetoclax, Sonrotoclax.
Oxidative phosphorylation—TCA cycle	HIF, c-myc, p52, EGFR, ras/BRAF, AKT, mTOR	ATP production. Thermogenesis. Adaptive responses.	PDH-inhibitors (e.g., devimistat). Metformin.
Glycolysis and the Warburg effect	HIF, AKT, mTOR, LDHA, CA9, GLUTs, SLC5A, viral genes (EBV, HPV)	ATP production. Production of carbon skeletons for lipid. Amino-acid and nucleotide biosynthesis.	HIF-inhibitors (e.g., PX-478, chetomin, niclosamide). LDHA-inhibitors (e.g., GSK2837808A, FX11, Oxamate, and natural compounds like Galloflavin). GLUT inhibitors (WZB117, BAY-876, Pan-GLUT inhibitor DRB18, HY-139605). SLC5A1/SLC5A2 inhibitors (e.g., canaglifozin, dapaglifozin). CA9-inhibitors (e.g., SLC-0111, methazolamide, acetazolamide). PDH-kinase inhibitors (e.g., Dichloroacetate).
The pentose phosphate pathway	Hexokinase, G6PD, wnt, c-myc, mTOR, Nrf2/EGFR, Src	NADPH provision. Biosynthesis of nucleotides, fatty acids and cholesterol.	Sanil-repressors (e.g., DHEA). G6PD-inhibitors (e.g., resveratrol, polydatin).
Amino-acid metabolism	mTOR, c-myc, EGFR, Kras, amino-acid transporters (ASCT2)	ATP production. NADPH and FADH2 synthesis.	ASCT2/SLC1A5 transporter inhibitors. Serine hydroxymethyltransferase inhibitors.
Lipid metabolism	c-myc, Kras, c-jun, HIF, citrate transporter, ACSL1 and FASN synthase, ACLY	Lipid synthesis. ATP production.	ATP Citrate Lyase inhibitors (e.g., BMS303141).
Autophagy and Lipophagy	ULK1, MAP1LC3, HIF, AKT, p52, mTOR, bcl-2, PLNs	Provides substrates for energy and building blocks.	Chloroquine. ULK-inhibitors (e.g., SBI-0206965, ULK-101, MRT68921). Glycogen synthase kinase 3 inhibitor (GSK-3beta).

**Table 2:** The main metabolic pathways active in cancer cells that can be targeted to enhance radiotherapy-induced anti-tumor immune responses and promote post-irradiation tumor clearance.

Metabolic Pathway	Therapeutic Targets to Enhance/Unblock Anti-Tumor Immune Response	Targeting Drugs
Oxidative stress response	Proteasome. NF-kb. IKK.	NF-κB inhibitors (e.g., Helenalin, curcumin, resveratrol). Proteasome inhibitors (e.g., Bortezomib, MG132). IKK complex inhibitors, (e.g., BAY 11-7082, SR 12343).
Glycolysis and the Warburg effect	HIFs. LDHA. GLUT. SLC5A. CA9. PDH-kinase.	HIF-inhibitors (e.g., PX-478, chetomin, niclosamide). LDHA-inhibitors (e.g., GSK2837808A, FX11, Oxamate, and natural compounds like Galloflavin). GLUT inhibitors (WZB117, BAY-876, Pan-GLUT inhibitor DRB18, HY-139605). SLC5A1/SLC5A2 inhibitors (e.g., canaglifozin, dapaglifozin). CA9-inhibitors (e.g., SLC-0111, methazolamide, acetazolamide). PDH-kinase inhibitors (e.g., Dichloroacetate).
ATP and adenosine pathway	P2X7 purinergic receptors. Ectonucleotidases CD73, CD39.	AZ10606120. Sanil-repressors (e.g., DHEA). AB-680 (and AMPCP. Suramine.
Amino-acid metabolism	IDO1, TDO2. ASS1.	Epacadostat, Indoximod, BMS-986205. Spinosine A derivatives.
Autophagy	ULK1. LC3. Autophago-lyosomal fusion. Lysosomes.	ULK inhibitors (e.g., SBI-0206965, ULK-101, MRT68921). Chloroquine.

**Table 3:** Diagnostic workup for tumor molecular and immunological characterization to establish a foundation for subsequent therapeutic interventions in the context of M.I.A.R (stratified by readiness for clinical application).

Essential Biomarkers for Guiding M.I.A.R.		
**A. Biomarkers already in clinical use** Validated tests (companion or established diagnostic or prognostic tests) that directly inform diagnosis, staging, or selection of an approved therapy.		
**Oncogene profiling**	Next-generation sequencing	EGFR mutations (exon 19 deletions, L858R, exon 20 insertions). ALK rearrangements/fusions. ROS1 rearrangements/fusions. BRAF V600E mutations. KRAS G12C mutations. MET exon 14 skipping and amplification. RET fusions. HER2 (ERBB2) mutations. NTRK1/2/3 fusions.
	In situ hybridization/FISH	HER2 amplification. ALK gene rearrangements. ROS1 gene rearrangements. EWSR1 rearrangements. 1p/19q co-deletion. HPV DNA or mRNA ISH. EBV. HBV/HCV status.
**Proliferation**	Immunohistochemistry	Ki-67/MIB-1 index.
**Tumor-suppressor/apoptosis**	NGS/Immunohistochemistry	TP53 mutation. Nuclear p53 expression.
**DNA-damage-repair/homologous-recombination status**	NGS/genomic assay	BRCA1/BRCA2 mutation (germline and somatic). HRR panel (e.g., ATM, PALB2, RAD51C/D, CHEK2, FANC genes). HRD/genomic-instability score (LOH, TAI, LST).
**Immune-checkpoint molecules**	IHC/NGS/PCR	PD-L1 (tumour proportion score/combined positive score). Mismatch-repair status: MSI-H/dMMR (MLH1, MSH2, MSH6, PMS2). Tumour mutational burden (TMB ≥ 10 mut/Mb).
**Functional imaging**	PET/CT	18F-FDG PET/CT (incl. total-lesion glycolysis for response assessment).
**B. Biomarkers extensively validated and studied in translational research** Hallmark-based markers with mechanistic rationale validated extensively in translational studies; not currently used to guide clinical research or routine treatment decisions.		
**Angiogenesis**	Immunohistochemistry	High vascular density. VEGF and VEGF-receptor expression.
**Apoptosis**	NGS/IHC/PET-CT	PARP1 gene alterations. Bcl-2 overexpression. IAP, XIAP overexpression. 18F-FluorThanatrace PET (PARP-1 target-directed imaging).
**Hypoxia**	IHC/PET-CT	Low vascular density. HIF. CA9. 18F-Misonidazole PET.
**Glycolysis**	IHC/Serum-Plasma ELISA	GLUT expression. LDHA expression. PDH-kinase expression. Hexokinase expression. MCT expression. Phosphorylated AKT expression. LDH isoenzyme analysis.
**Autophagy**	IHC/Confocal microscopy	LC3A,B. LAMP1,2. PLINs. LC3/LAMP co-localization.
**Stem cell**	Immunohistochemistry	CD44/CD25. CD133.
**Senescence**	Immunohistochemistry	SenTraGor^TM^.
**Immune** **-** **checkpoint molecules**	Immunohistochemistry	PD-1. CD80/CD86. TIM1. LAG3.
**Immunosuppressive microenvironment**	Immunohistochemistry	IDO1/TDO2. ASS1. Ectonucleotidases CD73, CD39.
**Tumour-infiltrating** **lymphocytes**	IHC/multiplex IF/PET-CT	TIL density. CD8. CD4, CD25, FOXP3. CD56. 18F-AraG PET-CT.
**Tumour** **-infiltrating macrophages**	Immunohistochemistry	CD68/CD44/SIRPα.
**Systemic immunity**	Flow cytometry	CD4/CD8 T-cell ratio. CD4/CD25/FOXP3 regulatory T-cells. CD8/TIM3, CD8/LAG3, CD8/PD-1, CD8/TIGIT exhaustion T-cell markers.

## Data Availability

No new data were created or analyzed in this study. Data sharing is not applicable to this article.
